# Revisiting Theories of Humidity Transduction: A Focus on Electrophysiological Data

**DOI:** 10.3389/fphys.2017.00650

**Published:** 2017-09-05

**Authors:** Harald Tichy, Maria Hellwig, Wolfgang Kallina

**Affiliations:** Department of Neurobiology, Faculty of Life Sciences, University of Vienna Vienna, Austria

**Keywords:** moist cell, dry cell, mechanical hygrometer, psychrometer, evaporation detector, insects

## Abstract

Understanding the mechanism of humidity transduction calls for experimental data and a theory to interpret the data and design new experiments. A comprehensive theory of humidity transduction must start with agreement on what humidity parameters are measured by hygroreceptors and processed by the brain. Hygroreceptors have been found in cuticular sensilla of a broad range of insect species. Their structural features are far from uniform. Nevertheless, these sensilla always contain an antagonistic pair of a moist cell and a dry cell combined with a thermoreceptive cold cell. The strategy behind this arrangement remains unclear. Three main models of humidity transduction have been proposed. Hygroreceptors could operate as mechanical hygrometers, psychrometers or evaporation detectors. Each mode of action measures a different humidity parameter. Mechanical hygrometers measure the relative humidity, psychrometers indicate the wet-bulb temperature, and evaporimeters refer to the saturation deficit of the air. Here we assess the validity of the different functions by testing specific predictions drawn from each of the models. The effect of air temperature on the responses to humidity stimulation rules out the mechanical hygrometer function, but it supports the psychrometer function and highlights the action as evaporation rate detector. We suggest testing the effect of the flow rate of the air stream used for humidity stimulation. As the wind speed strongly affects the power of evaporation, experiments with changing saturation deficit at different flow rates would improve our knowledge on humidity transduction.

## Introduction

Insects are continually challenged to achieve and maintain optimal functions as ambient humidity changes. In addition to metabolic adaptations, most insects improve their survival abilities by humidity preference and avoidance responses. These behaviors make the existence of hygroreceptors very likely. The structural and the electrophysiological properties of hygroreceptors have been studied in only a few insect species. These hygroreceptors are associated in antagonistic pairs of a moist and a dry cell in the same sensillum with a thermoreceptive cold cell. The mechanism by which humidity stimulates the moist and dry cells remains controversial. Three main models of humidity transduction have been proposed, namely in which hygroreceptors operate as mechanical hygrometers, psychrometers or evaporation detectors (Tichy and Loftus, 1996; Steinbrecht, 1999; Tichy and Gingl, 2001). These models imply very different stimulus-response relationships, which give rise to some confusion concerning the definition of the adequate humidity stimulus. The moist cell and the dry cell appear to be bimodal in that their responses to humidity strongly depend on temperature. Either modality can be changed independently of the other, but both are related in some way to the amount of moisture in the air and to its influence upon evaporation. Here we challenge the hygro-mechanical function most favored for humidity transduction and provide further arguments for an evaporative function of hygroreceptors. This brief account discusses the validity of the three main models of humidity transduction (mechanical hygrometer, psychrometer and evaporation rate detector) by determining whether specific predictions based upon them are indeed observed in electrophysiological responses. We first provide short background information of the ways of expressing and measuring humidity.

## Measures of humidity

**Figure 1:**
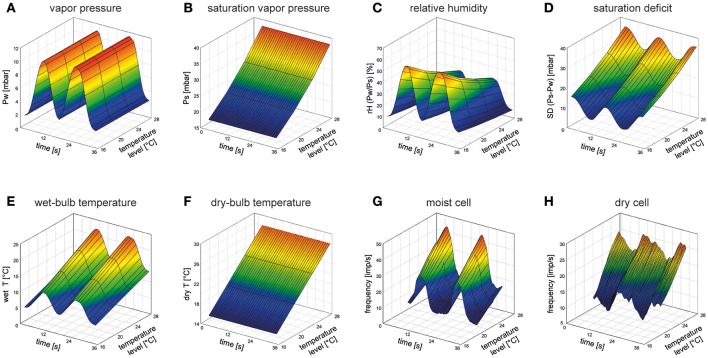
3D-mesh plots of the effects of air temperature on different ways of expressing humidity and the responses of a moist cell and a dry cell during two periods of slowly oscillating changes in water vapor pressure. **(A)** Humidity stimulation consisted of two consecutive periods of constant-amplitude oscillations in vapor pressure (0–15 mbar) at four different temperature levels of 21.0, 22.8, 24.7, and 26.8°C. **(B)** Saturation vapor pressure at the same temperature levels. Constant-amplitude oscillating change in vapor pressure in *A* produces, with rising temperature, continuously deceasing oscillations in relative humidity **(C)**, but continuously increasing oscillations in both saturation deficit **(D)** and wet-bulb temperature **(E)**. Dry-bulb temperature as a function of air temperature **(F)**. **(G,H)** Impulse frequency of a moist cell and a dry cell recorded simultaneously from the same sensillum on the cockroach's antenna during oscillations in vapor pressure at the different temperature levels (21.0, 22.8, 24.7, 26.8°C) illustrated in **(A)**. With rising temperature, the oscillations in impulse frequency of the moist and dry cells shift upwards on the frequency scale. Impulse frequency (*F*, impulses/s) was calculated from running averages of three consecutive 0.5-s intervals (Tichy and Kallina, 2013). Water vapor density of the stimulating air stream (flow rate, 2.5 m/s) was measured at a rate of 100 Hz by an UV-absorption hygrometer (K 20, Campbell Scientific) and air temperature (dry-bulb temperature, *dry T*) was measured within ±0.03°C by a small thermistor (250 × 400 μm; Fenwall Electronics, BC 32 L1). Based on the digitized signals of the hygrometer and the thermistor, the vapor pressure (*Pw*) and the relative humidity (*rH*) were monitored offline. The saturation water vapor (*Ps*), the saturation deficit (*SD*) and the wet-bulb temperature (*wet T*) were calculated using the Vaisala Humidity Calculator, a web-based software tool.

Humidity refers to the amount of evaporated water in the air and is defined as the partial pressure exerted by the evaporated water vapor on the total pressure of the air. The water vapor pressure is not affected by air temperature (Figure 1A). Increasing air temperature increases the kinetic energy of the molecules in the air, but not the concentration of the vapor molecules. The more kinetic energy in the air, the more water can be evaporated, and the more water vapor is required for saturation of the air. The saturation vapor pressure increases as air temperature increases (Figure 1B). The relative humidity is the ratio between the water vapor actually in the air and the saturation vapor pressure (Figure 1C), indicating how close the air is to saturation. Therefore, the relative humidity is not a direct measure of the amount of water vapor in the air. As a ratio, the relative humidity is dimensionless; if it is used as a humidity parameter, then—for completeness—the air temperature must be supplied with it. The saturation deficit, in contrast, is a measure of humidity that is expressed in vapor pressure units. It is the difference between the theoretical water pressure at saturation and actual vapor pressure in the air being measured at the same temperature (Figure 1D). While an increase in the relative humidity at constant vapor pressure is correlated with decreasing air temperature (Figure 1C), the saturation deficit increases with increasing air temperature (Figure 1D). Thus, the relative humidity is inversely related to air temperature (Figure 1C) and the saturation deficit is directly related to air temperature (Figure 1D). In contrast to the relative humidity, the saturation deficit integrates in a single value the effects of both temperature and humidity (or dryness) of the air on the evaporation rate.

Evaporation of water can also be measured psychrometrically by the degree of cooling at the evaporating surface. As the water molecules escape, they take kinetic energy with them, leaving the surface with a diminished total kinetic energy. If all the latent heat of vaporization has been supplied to the air, then this temperature is known as the wet-bulb temperature: the lowest temperature to which the surface can be cooled by evaporation of water (Figure 1E). It is the temperature that would be taken up by a thermometer bulb kept moist by a thin wet covering. In order to determine the temperature depression due to the cooling effect of the evaporating water, a second temperature reading is needed from a thermometer with a dry surface (dry-bulb temperature) indicating air temperature. Raising air temperature by adding (sensible) heat increases both the dry-bulb (Figure 1F) and the wet-bulb temperature (Figure 1E), but there is no change in the evaporated water in the air. A system functioning as a psychrometer would require two temperature-sensitive hygroreceptors, one beneath a dry surface and unaffected by cooling and another with a wet surface cooled by evaporation.

## Electrophysiological identification

The most commonly used stimuli in studies on humidity transduction were rapid changes in the humidity of an air stream flowing over the antennae (Yokohari and Tateda, 1976; Yokohari, 1978; Itoh et al., 1984; Tichy, 1987; Tichy and Loftus, 1996). However, transient humidity changes were too fast in order to measure the rate with which humidity was changing at the sensillum. The problem was solved by applying slow and continuous changes in humidity, at rates low enough so that the humidity of the air stream is equivalent to that of the sensillum and, furthermore, the sensillum's moisture content is allowed to reach equilibrium with that of the air stream (Tichy, 2003). Such experiments have been performed so far on cockroaches, stick insects and honey bees (Tichy, 2003; Tichy and Kallina, 2010, 2013, 2014). Here, the issue is being taken up again on the cockroach because quite a bit of work has been done on this insect already.

When the water vapor pressure is made to oscillate smoothly during 8 to 10 s periods and at amplitudes of 10 to 12 mbar (Figure 1A), the discharge rate of the moist cell increases while the vapor pressure rises and decreases as it falls (Figure 1G). Correspondingly, contrary effects are elicited in the dry cell (Figure 1H). As the 3-D mesh plots indicate, the higher the temperature level of the oscillating changes in vapor pressure, the stronger the oscillating responses of the moist and dry cells. The dependence of both cells on air humidity and temperature is not a question of the adequate stimulus in the sense of the modality in which the smallest change elicits a response. Rather, it is a question of whether changes in a single parameter can explain the reactions to all the changes recorded.

## Mechanical hygrometer model

A mechanical mode of action views the cuticular wall of the sensillum as a hygro-mechanical transducer. Humidity-dependent shrinking due to water loss and swelling due to water uptake is believed to alter the geometry of the cuticular wall that, in turn, leads to deformation of the dendritic membranes and voltage changes across them (for reviews see Tichy and Loftus, 1996; Steinbrecht, 1999; Tichy and Gingl, 2001). Mechanical hygrometers such as hair hygrometers make use of a change in length which is proportional to the relative humidity. When the water vapor remains constant (Figure 1A), the relative humidity decreases with rising temperature (Figure 1C). However, the impulse frequency of the moist and dry cell to fluctuations in humidity increases with rising temperature (Figures 1G,H). The positive temperature coefficient of the hygroreceptor's responses to changes in the relative humidity contradicts a hygro-mechanical transducer. Moreover, it is difficult to imagine how the sensillum wall is so hygroscopic that it can withdraw water vapor from the air in quantities large enough to produce a graded mechanical effect on the dendrites without being affected by the water inside the sensillum. Thus the hygrometer model seems beset with insurmountable difficulties.

## Psychrometer model

Psychrometers measure air humidity by means of a wet-bulb and a dry-bulb thermometer. The wet-bulb temperature will be lower than the dry-bulb temperature due to the cooling effect (loss of latent heat) of water evaporating from its surface. Rising air temperature (sensible heat) increases both the wet-bulb and dry-bulb temperatures (Figures 1E,F), but the latent heat of the air remains constant. The increased response of the moist and dry cell to humidity fluctuations with rising temperature (Figures 1G,H) is in line with a wet-bulb thermometer (Figure 1E). The moist-cell's impulse frequency corresponds with the wet-bulb temperature (with a decreasing cooling effect due to decreasing evaporation) and the dry cell responds antagonistically to the moist cell. The thermoreceptive cold cell could be assumed to measure the dry-bulb temperature. The cold cell is located in the same sensillum with the most and dry cell and shares functionally the same receptive field with the two hygroreceptors (Tichy, 1987). Nonetheless, the cold-cell's power to discriminate temperature levels is rather poor; two temperature levels must differ by 0.9°C in order to identify one of them as being higher than the other (Tichy and Loftus, 1987). Furthermore, it is very difficult to imagine how a wet and a dry surface could be maintained by the sensillum during fluctuating changes in humidity. This would mean measuring the wet-bulb temperature during cooling by evaporation while keeping the dry-bulb temperature unaffected by adjoining evaporation cooling.

## Evaporation model

The loss of water by evaporation is directly proportional to the saturation deficit of the air. Rising air temperature increases the saturation deficit (Figure 1D) when the vapor pressure remains constant (Figure 1A). The increased response of the moist and the dry cell to fluctuations in humidity with rising temperature (Figures 1G,H) fit well to the temperature dependence of the saturation deficit (Figure 1D). The antagonistic responses suggest that the moist cell is excited by decreasing evaporation rates, the dry cell by increasing rates. As with the psychrometer model, a system functioning as an evaporation rate detector requires that lymph inside the sensillum moves toward the outside, where the water content is exposed to controlled evaporation in ambient air. Evaporation may lead to a quantitative concentration change of the lymph, changes in osmotic pressure or mechanical stress in the dendrites of the receptor cells.

Thus, transduction may involve chemical or mechanical mechanisms. Nonetheless, an evaporation controller that relies on concentration sensors might be unstable because chemical concentration is affected by multiple factors. Evaporation acting by fluid shear stress, pressure across, or tension within the dendritic membranes may create mechanical forces that deform membranes—as has been suggested for the mechanical hygrometer model. By contrast, the mechanosensory transduction stimulated by evaporation requires fine-tuning of the transducing ion channels. Because the moist cell and the dry cell discharge continuously during upward and downward changes in the saturation deficit, a receptor current must continuously flow when the evaporation rate is in equilibrium with ambient humidity. The current will be modulated between maximum and minimum values, and the sign will be opposite in the moist and dry cell. Conductivity of both antagonists must account for this symmetric change in receptor current as well as for its maintenance. A simple mechanism described for mechanosensitive channels is based on variation of the membrane capacitance (Petrov and Usherwood, 1994). Stretching a cell membrane and increasing its area (simultaneously decreasing its thickness) will increase the membrane capacitance (a dynamic measure of membrane surface area; approximately 1 μF per 0.5–1 cm^2^ surface area; Apodaca, 2002). Subjecting the membrane to oscillating deformations will modulate its membrane capacitance and the flow of the capacitance current.

## Conclusions and future prospects

Electrophysiological studies on the cockroach revealed a dependence of the responses of the moist and the dry cell to slowly fluctuating changes in vapor pressure on the temperature level at which the humidity changes are carried out (Figures 1G,H). The positive temperature coefficient has implications for the validity of the humidity transduction models. Expressing the fluctuation in vapor pressure (Figure 1A) as fluctuations in the relative humidity (Figure 1C), the resulting negative temperature coefficient excludes a mechanical hygrometer function. The positive temperature coefficient of the wet-bulb temperature (Figure 1E) supports a psychrometric measurement. However, the close proximity of a “wet-surface” and a “dry-surface” on the same sensillum make it difficult to understand how the dry air temperature could be taken without being affected by evaporative cooling. The positive temperature coefficient of the saturation deficit (Figure 1D) highlights an evaporative function. A key experiment for testing a specific prediction drawn from the evaporation model would be to alter the flow rate of the stimulating air stream. Flow rate drastically affects evaporation power. Thus, experiments with slow and continuous changes in the saturation deficit at different flow rates and temperatures would assess the validity of an evaporative function. From the small amount of information currently available it is not possible to relate the type of response of the hygroreceptive sensory cells with any locomotor reactions which result in the insect aggregation in preferred humidity zones. Further detailed studies of these aspects in insect species known to inhabit different humidity environments would be highly desirable.

## Author contributions

All authors contributed to data analysis, interpretation of results, writing the manuscript and designing the figures.

### Conflict of interest statement

The authors declare that the research was conducted in the absence of any commercial or financial relationships that could be construed as a potential conflict of interest.
